# Data-driven analysis and prediction of stable phases for high-entropy alloy design

**DOI:** 10.1038/s41598-023-50044-0

**Published:** 2023-12-18

**Authors:** Iman Peivaste, Ericmoore Jossou, Ahmed A. Tiamiyu

**Affiliations:** 1 Department of Mechanical and Manufacturing Engineering, University of Calgary, Calgary, Alberta, T2N 1N4, Canada; 2 Nuclear Science and Technology Department, Brookhaven National Laboratory, Upton, NY 11973 USA; 3 Present Address: Department of Nuclear Science and Engineering, Massachusetts Institute of Technology, Cambridge, MA 02139 USA

**Keywords:** Materials science, Computational methods

## Abstract

High-entropy alloys (HEAs) represent a promising class of materials with exceptional structural and functional properties. However, their design and optimization pose challenges due to the large composition-phase space coupled with the complex and diverse nature of the phase formation dynamics. In this study, a data-driven approach that utilizes machine learning (ML) techniques to predict HEA phases and their composition-dependent phases is proposed. By employing a comprehensive dataset comprising 5692 experimental records encompassing 50 elements and 11 phase categories, we compare the performance of various ML models. Our analysis identifies the most influential features for accurate phase prediction. Furthermore, the class imbalance is addressed by employing data augmentation methods, raising the number of records to 1500 in each category, and ensuring a balanced representation of phase categories. The results show that XGBoost and Random Forest consistently outperform the other models, achieving 86% accuracy in predicting all phases. Additionally, this work provides an extensive analysis of HEA phase formers, showing the contributions of elements and features to the presence of specific phases. We also examine the impact of including different phases on ML model accuracy and feature significance. Notably, the findings underscore the need for ML model selection based on specific applications and desired predictions, as feature importance varies across models and phases. This study significantly advances the understanding of HEA phase formation, enabling targeted alloy design and fostering progress in the field of materials science.

## Introduction

The field of alloy design and optimization has witnessed significant progress, transitioning from simple compositions to complex alloys with improved mechanical and physiochemical properties. A new class of alloys called High-Entropy Alloys (HEAs) has revolutionized alloy development by focusing on the largely unexplored central regions of multi-element phase diagrams, where concentrated amounts of four or more alloying elements exist, and there is no obvious single base element. This novel approach paves the way for exploring countless alloy systems by harnessing the vast combinatorial potential of elements in the periodic table. Besides, it presents a remarkable opportunity to discover materials that exhibit unprecedented combinations of structural and functional properties^1–6^. HEAs have demonstrated remarkable characteristics such as high-temperature resilience, specific strength (lightweighting), corrosion resistance, and radiation tolerance; these make them highly promising for various applications^7–12^. With proper design and development, HEAs hold the potential to introduce groundbreaking capabilities in mechanical, thermoelectric, and magnetic properties that were previously unattainable in traditional alloys^13–16^.

The surge in HEA research, particularly over the last decade, can be attributed to the seminal work of Yeh et el.^17^ and Cantor et al.^18^ in 2004, which has broadened the field of alloy design with an emerging sub-track in HEA. After the initial design of HEAs, the constraints of equiatomic composition were relaxed, allowing for a broader range of concentrations from 5 to 35 atomic percent (at.%) for each constituent element^19^.

Despite the recent progress, phase prediction remains difficult and complex; hence, the accurate determination of the phase formation remains paramount when designing novel HEAs with targeted properties, as it is intricately tied to specific material characteristics^20–23^. Phases in HEAs can be a solid solution (SS), intermetallic (IM), amorphous (AM), and a mixture of these phases (SS + IM and SS + AM)^24^. While SS HEAs primarily exist in face-centered cubic (FCC), body-centered cubic (BCC), or hexagonal closed pack (HCP) structures, there are limited works on HCP phase^25^, and would not be considered in this study due to the limited dataset in literature. Under certain conditions, multiple phases can co-exist in SS HEAs. For example, some HEAs can undergo spinodal decomposition upon quenching from high temperatures, resulting in a nanoscale separation into two solid solutions^1^. At the very early stage of HEA exploration, HEAs garnered significant attention primarily due to the unexpected emergence of a single-phase solid solution (SSS)^5^. However, in the present day, approximately two decades following the initial HEA discovery, the field has experienced substantial growth, and studies have expanded beyond the development of the homogeneous SS phase; IM and AM phases are now being explored. Furthermore, efforts are underway to develop HEAs with fewer elements, aiming to optimize their properties^26^. In addition, innovative microstructures are being designed, incorporating multiple phases to precisely tailor the characteristics of HEAs for targeted applications^27,28^. The increasing interest in the field of HEAs is driven by the increasing demand for materials in the emerging clean energy domain and opportunities to extend the lifetime of alloys in extreme or harsh environments.

Traditionally, the design and fabrication of HEAs relied on trial and error, empirical guidelines that sometimes fail, and chance discoveries^14,29,30^. However, efficient computational techniques have emerged, and they have since offered a more reliable alloy-design pathway, rather than relying on costly trial and error or serendipity. By leveraging advanced algorithms and simulations, computational materials design has paved the way for a more systematic and efficient exploration of chemically complex materials^31–35^. Several simulations at different lengths and time scales have been proposed to accurately predict the thermodynamics of phase stability in these compositionally-complex alloy systems. Traditional simulation methods rooted in classical physics encounter significant limitations right from the initial stages, particularly in accurately predicting phase equilibria in high-dimensional systems. Oftentimes, the computational cost can be prohibitive, restricting their application to smaller systems or making it impractical to explore complex, high-dimensional alloy systems thoroughly. Furthermore, these methods struggle to provide reliable simulations for non-equilibrium microstructures and associated properties, further highlighting their shortcomings^31,36,37^.

Over the past decade, there has been an extraordinary surge in the prominence of machine learning (ML), and it has made a significant impact across numerous scientific disciplines. ML is a branch of artificial intelligence (AI) that involves training algorithms to analyze complex patterns^12,38,39^. The far-reaching influence of AI and ML has extended their application and relevance to a wide array of scientific fields, transcending the boundaries of traditional computational approaches. The capabilities of ML models are continuously enhanced by the accessibility of powerful computing resources, advanced algorithms, and vast amounts of data obtained from experiments or computations. These qualities position ML as a highly promising tool to tackle the challenges faced by the theoretical modeling of HEAs. ML plays a multifaceted role in the development of HEAs, as outlined in relevant review papers^20,32,40–43^. It serves various purposes, including predicting stable phases (microstructure) and properties, accelerating simulations, and extracting underlying physical principles from the complex chemical structure of HEAs. Notably, since 2018, many studies have explored the application of ML in predicting phases, encompassing various aspects. Some studies focused on distinguishing whether a SS is a single phase or a non-single phase^30^; other works delved into classifying SSS vs dual phase (SS+IM)^44^; SSS vs IM vs AM^45–48^; SSS vs IM vs dual phase (SS+IM)^49,50^; SSS vs IM vs AM vs dual phase (SS+IM)^37,51–53^; SSS vs IM vs AM vs dual phase (SS+AM)^54^; FCC vs BCC vs dual phase (BCC+FCC)^55–58^; FCC vs BCC vs dual phase (BCC+FCC) vs IM^59,60^; FCC vs BCC vs HCP vs IM vs others^61,62^; FCC vs BCC vs IM vs AM^63^; FCC vs BCC vs HCP vs Multiphase^64^. The existing body of literature highlights the significance of predicting whether a SS is a single phase or a mixture of solid solutions with other phases. However, recent studies have made efforts to further enhance the precision of phase prediction within a SSS. This is an important advancement, as different phases within a SS exhibit distinct mechanical properties^65,66^. A seminal paper by Yeh^17^ demonstrated that altering the phase composition from FCC to BCC in the AlxCoCrMnNi solid solution significantly increased its hardness by a factor of 7 when the aluminum content was increased from 0.1 to 3 wt.%. IM and AM phases, in particular, hold great importance in this field as dual-phase solid solutions with IM mixtures offer promising properties. HEAs with IM phases have high thermal stability, strength and ductility especially when combined with SS^37,67,68^. Achieving the desired strength and toughness relies on effectively balancing the combination of ductile and brittle phases^27^. Meanwhile, the inclusion of AM phases in HEAs facilitates the development of materials with notable characteristics such as large elastic strain, enhanced corrosion resistance, and improved wear resistance^69^. However, identifying IM or AM phases within HEAs poses challenges due to their lower sensitivity to empirical parameters^55^. Consequently, the accurate identification and characterization of mixtures involving SS, IM, and AM phases have not been extensively addressed. To solve this problem, unique features are required to accurately predict these phases. The need for precise phase identification is therefore crucial, as the mechanical properties of a mixture containing FCC and IM phases, for instance, differ significantly from those of a mixture containing BCC and IM phases. Furthermore, although there have been significant contributions to the use of ML for phase prediction in HEAs, most of these studies relied on relatively small datasets containing fewer than 1000 experimental records with similar features. The handful of studies that employed larger datasets with over 2000 data points, such as^54,59,61,70^, are either limited in terms of the number of elements and phases considered or experience unequal distribution of samples across different classes or categories. These limitations highlight the need for studies that can address a wider range of elements and phases while ensuring a balanced dataset.

This study aims to develop a data-driven method for:

- Identifying the key factors influencing the presence of specific phases.
- Determining the most effective approach for phase prediction through employing multiple ML algorithms and assessing their performance.
- Predicting the most common phases in HEAs and their combinations, encompassing a total of 11 categories, from a balanced dataset consisting of 50 different elements.
- Assessing the effect of incorporating IM and AM phases, on the accuracy of ML models for phase prediction in HEAs, and examining how the inclusion of IM and AM phases influences the performance of ML algorithms.
- Investigating the variations in feature importance when different phases are considered; highlighting the shifting significance of key features in accurately predicting the diverse range of phases encountered in HEAs.

To achieve these aims, an extensive dataset of approximately 5692 records, covering all possible phases in HEAs, including BCC, FCC, IM, AM, and their combination is utilized to build the models. The accuracy of the models for each phase category is thoroughly analyzed, and relevant descriptors or features contributing to phase prediction are identified. The study also addresses the challenge of an imbalanced dataset by employing data augmentation techniques. The impact of these techniques on the overall accuracy of the model is evaluated, ensuring its reliability and robustness in handling imbalanced data scenarios in the field of HEAs.

## Results

A total of 11,252 experimental observations of HEAs were collected and categorized into 11 distinct phases or phase combinations, including BCC, FCC, BCC+FCC, IM, BCC+IM, FCC+IM, BCC+FCC+IM, AM, BCC+AM, FCC+AM, and BCC+FCC+AM. To facilitate analysis and modeling, we employed elemental properties, empirical rules, and a set of principles and formulas to generate features for each observation. Subsequently, the collected data underwent a comprehensive data-cleaning process to ensure data quality and reliability. This encompassed various tasks such as handling missing values, removing duplicates, standardizing formats, dealing with outliers, and resolving inconsistencies. These steps were undertaken to enhance the data’s quality, integrity, and reliability for developing accurate predictive ML models for HEAs. Following the data scrubbing process, a set of 5,692 observations with 12 features were obtained, which constituted the cleaned and refined dataset ready for the machine learning task at hand. However, an issue arose due to the imbalanced distribution of data across the different categories. To mitigate this, we employed data augmentation techniques to generate synthetic data, thereby achieving a more balanced representation of the various categories. This step was crucial to ensure a robust and reliable analysis of the HEAs dataset.

### Exploring composition trends to identify phase formers from dataset

The ability to comprehend complex information visually plays a fundamental role in constructing persuasive arguments, particularly within the field of materials science. This is because identifying and comprehending intricate rules and patterns from raw data can be inherently challenging. Visual aids provide invaluable insights that facilitate deeper exploration of data sets, ultimately leading to the discovery of valuable information. In the context of HEAs, most studies focus on investigating SS HEAs^4^. These SS HEAs typically consist of FCC, BCC, HCP structures, or a combination thereof. For this study, as mentioned earlier, HCP structures were excluded due to the limited availability of sufficient data on this particular structure. It is imperative to explore the factors that contribute to the formation of SS, IM, and AM HEAs. HEAs that exhibit an FCC structure are recognized for their favorable ductility. However, these alloys typically have lower strength. On the other hand, HEAs with a BCC structure can possess significantly greater strength, but this often comes at the expense of reduced ductility, especially under tension. Designing and manufacturing HEAs with desirable properties can be achieved through effective control of the formation of two or more phases. Therefore, studying the composition trends and prevalence of certain elements that influence the formation of SS, IM, and AM structures can provide valuable insights into the selection and design of HEAs.

**Figure 1:**
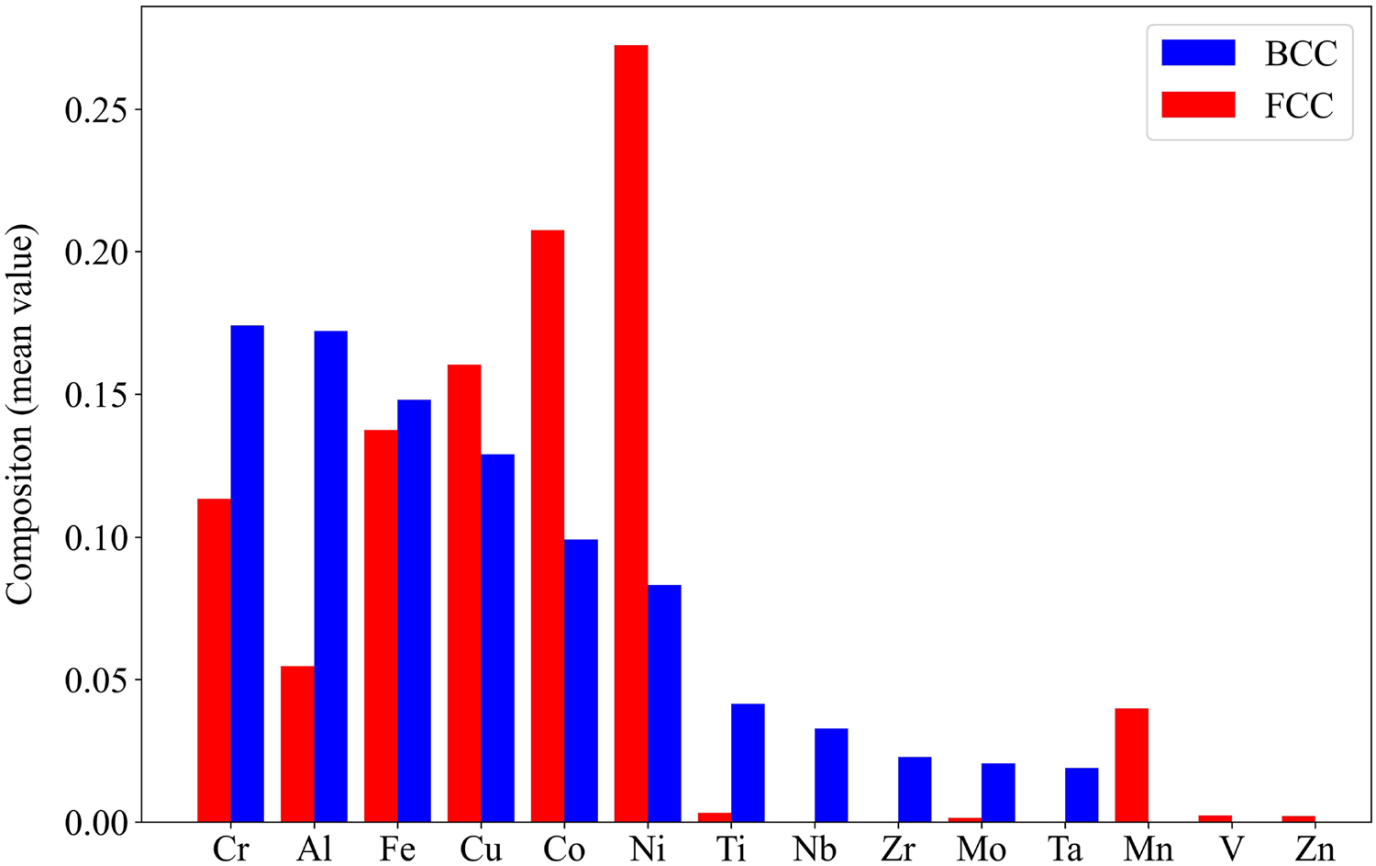
Comparison of mean composition values for commonly occurring elements in BCC and FCC phases. The figure highlights a higher presence of Ni and Co in the FCC phase, suggesting their role as strong FCC stabilizers. Conversely, Cr and Al show higher proportions in the BCC phase, indicating their influence as BCC stabilizing elements.

Fig. 1 presents a comparison of the mean composition values for the most frequently occurring elements in the BCC and FCC phases within the dataset. The figure shows that Ni and Co are more prevalent in the FCC phase. On average, Ni constitutes over 25 percent of the total composition in each data point belonging to the FCC phase, while Co accounts for approximately 20 percent. This observation suggests that Ni and Co serve as strong FCC phase formers or stabilizers. Additionally, elements such as Mn, V, and Zn promote the formation of the FCC phase. Conversely, higher amounts of Cr and Al are observed in the BCC phase, contributing about 17 percent of the composition in each alloy on average. Other BCC stabilizing elements identified in the dataset include Ti, Nb, Zr, Ta, and Mo. These results are also in agreement with the established FCC (austenite) and BCC (ferrite) formers or stabilizers in traditional alloys^71^.

**Figure 2:**
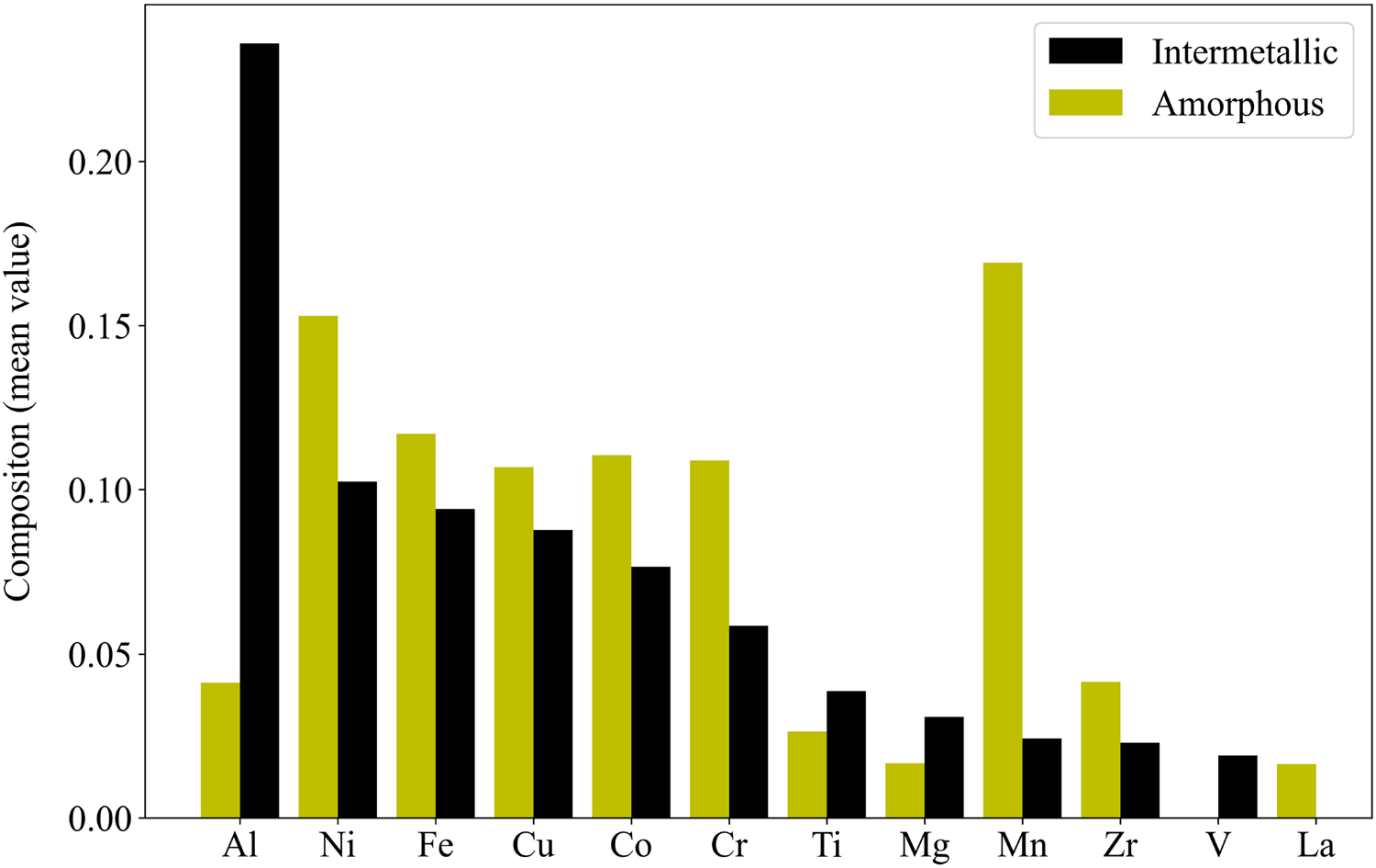
Comparison of mean composition values for commonly occurring elements in IM and AM phases. It highlights the higher presence of Al and Ni in the IM phase, suggesting their role as strong stabilizers. Conversely, Mn helps the formation of AM phase.

The average value of each of the top 12 elements in each record of IM and AM categories in our dataset is shown in Fig. 2. Our analysis highlights the pivotal role of Al in the formation of both the BCC and IM phases. Specifically, the average composition of Al in the BCC phase, as shown in Fig. 1 is approximately 17 percent, whereas its concentration surged to nearly 25 percent in the IM phase. This suggests that a higher concentration of Al promotes the formation of the IM phase in the alloy. Conversely, Mn emerges as the primary element in the AM category, with an average composition exceeding 15 percent. Interestingly, although Al played a significant role in the formation of both the BCC and IM phases, its contribution to the formation of AM is limited. Instead, Ni exhibited the second-highest proportion in the AM category, with an average content of 15 percent. These insights contribute to a better understanding of the factors influencing phase formation in HEAs and pave the way for tailored alloy design and improved material properties. In light of the dataset’s comprehensive and abundant nature, our analysis now focuses on evaluating the effectiveness of various ML algorithms that can be trained for phase prediction using this dataset.

### Evaluation of ML models for phase prediction in HEAs

HEAs are formed by the free-intermixing of multiple chemical species to form a compound with a preferred crystal structure and thermodynamically stable phases that are driven by thermodynamic considerations. However, it should be noted that not all combinations of chemical species produce single-phase HEAs. Instead, certain combinations lead to the segregation of distinct phases, including dual-phase SS HEAs, IM, AM structures, or even mixtures of dual or triple phases consisting of SS, IM, and AM regions. In this section, ML is used to predict different phases present in HEAs. Each ML model’s performance will be evaluated based on specific categories, and the significance or effectiveness of each feature will be ranked accordingly. By employing ML techniques, we aim to enhance the understanding of the phase formation in HEAs and develop reliable predictive models for future alloy design and synthesis.

The primary objective is to train several ML models to accurately predict various categories of HEAs. Initially, the focus is on three primary categories of SS HEAs: BCC, FCC, and the dual-phase combination of BCC and FCC. Recognizing the significance of AM phases in HEAs, we then expand the training to include predictions of AM structures and their potential mixture with SS HEAs and to further understand their (AM inclusion) impact on prediction accuracy. Although AM phases have received comparatively less attention in the field, their prediction holds immense value for the scientific community. Recent studies^72,73^ have reported significant phase stability of AM HEAs in extreme environments. Recent findings have also shown that AM HEAs have the ability to maintain their structural integrity under extreme conditions, offering new perspectives and opportunities for their application in various industries.

We also consider a similar analysis for IM phases, investigating the presence of pure IM phases and their potential combination with SS. We aim to assess how accurately the ML models could predict these scenarios in comparison to pure SS. Furthermore, to comprehensively evaluate the effectiveness of the models, we conduct tests to assess their accuracy in predicting all 11 possible combinations of phases in HEAs. By considering this wide range of phase combinations, we show that the models are capable of providing reliable predictions for the full spectrum of possible phases encountered in HEAs.

**Figure 3:**
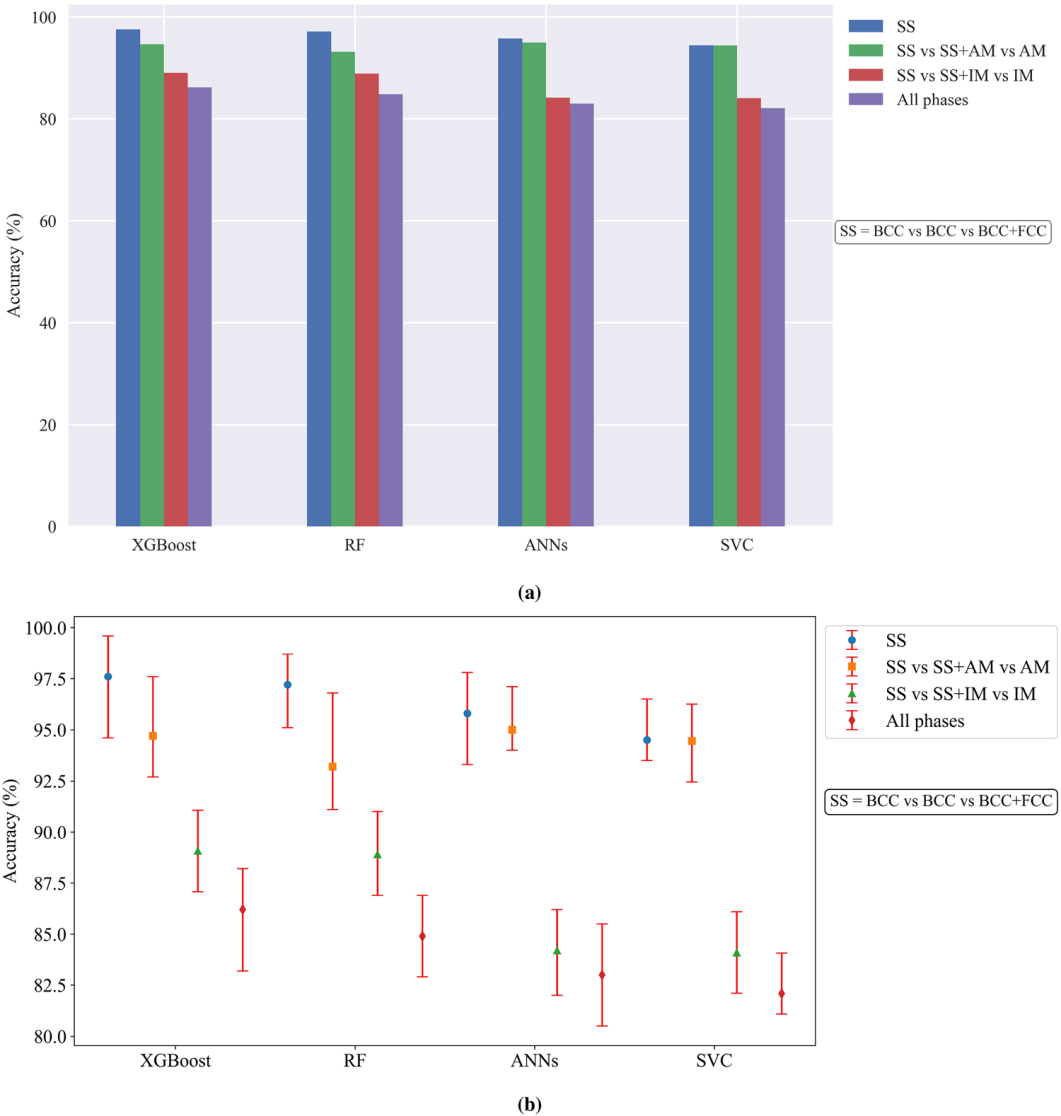
(a) Comparison of machine learning models for high-entropy alloy phase prediction, and (b) the comparison of accuracy with standard deviation under 5-fold cross-validation. Results show varying accuracy across different phase categories, with XGBoost consistently performing well and achieving the highest accuracy. SVC exhibits comparatively lower performance, while RF and ANNs demonstrate strong predictions for multiple phases. Accuracy slightly decreases when considering additional phases such as AM and IM, with XGBoost outperforming other models across all phase categories.

To evaluate the precision of our predictions, Fig. 3a offers a comprehensive assessment of various ML models using either the validation or test dataset in this research. Meanwhile, Fig. 3b presents a comparison of model accuracy through 5-fold cross-validation. This representation includes both average accuracy and standard deviation. To address the class imbalance, the training dataset was augmented, resulting in approximately 1500 data points for each category. The models are subjected to training and evaluation using identical datasets, ensuring a fair and consistent assessment. To provide a comprehensive evaluation of each model’s performance, including the assessment of standard deviation, a k-fold cross-validation is employed. In this study, we set the value of k to 5. This approach is particularly valuable when dealing with limited training data. Upon analyzing the results, it shows that when predicting SS phases, the tuned XGBoost and Random Forest (RF) models achieved the highest accuracy, an impressive 97%. Notably, this level of accuracy has been rarely observed in previous ML models applied to SS HEAs. It is worth mentioning that this exceptional accuracy was achieved while ensuring the removal of duplicates in the dataset, further emphasizing the robustness and reliability of the results. Artificial Neural Networks (ANNs) also exhibit strong performance, with an accuracy of 95%, correctly predicting outcomes for a significant portion of the testing data on BCC, FCC, and dual-phase BCC+FCC. In contrast, the support vector classifier (SVC) exhibits relatively lower, 94%, in comparison to the XGBoost in particular.

Expanding the number of categories to include the AM phase results in a slight drop in accuracy across the models. The ANNs maintain the highest accuracy at 95%, while both XGBoost and SVC achieve similar accuracy levels of approximately 94%. The RF model shows an accuracy of 93%. A similar analysis was performed for the IM phase by increasing the number of categories from 3 to 7, including all combinations with the IM phase. Interestingly, the addition of IM phases had a more pronounced impact on accuracy compared to the AM phases, suggesting that IM phases are less sensitive to the features used in the ML models. The IM-related categories led to an accuracy drop to less than 90 percent, with XGBoost and RF models both showing around 89% accuracy, while ANNs and SVC presented just above 84%. Finally, the comprehensive analysis encompassed all possible phases, incorporating 11 distinct categories involving SS, IM, AM, and their combinations. Among the ML models, XGBoost outperformed the others with an accuracy of 86%. The RF showed an accuracy of 85%, while ANNs and SVC achieved 83% and 82% accuracy, respectively. These findings demonstrate the varying performance of different ML models across various categories and the impact of expanding the number of categories on overall accuracy. XGBoost consistently showcases strong performance, particularly in predicting all phases, while SVC demonstrates relatively weaker performance across the different category expansions. The results emphasize the importance of considering the specific application and desired category predictions when selecting an appropriate ML model for HEA phase prediction.

There has been some concern regarding the use of synthetic data and its impact on the performance of ML models. Synthetic data that is not sufficiently realistic or representative of real data can reduce model accuracy or it might fail to capture the full diversity and complexity of real data. We find that the inclusion of synthetic data has a minimal effect on accuracy, with all models showing slight improvement, albeit less than a percent. This indicates that the quality of the generated data was high and effectively addressed the issue of biases in the dataset. The use of synthetic data, when appropriately generated, can enhance the reliability and robustness of ML models without significantly affecting their overall performance^74^.

### Feature importance in different ML models for phase prediction

**Figure 4:**
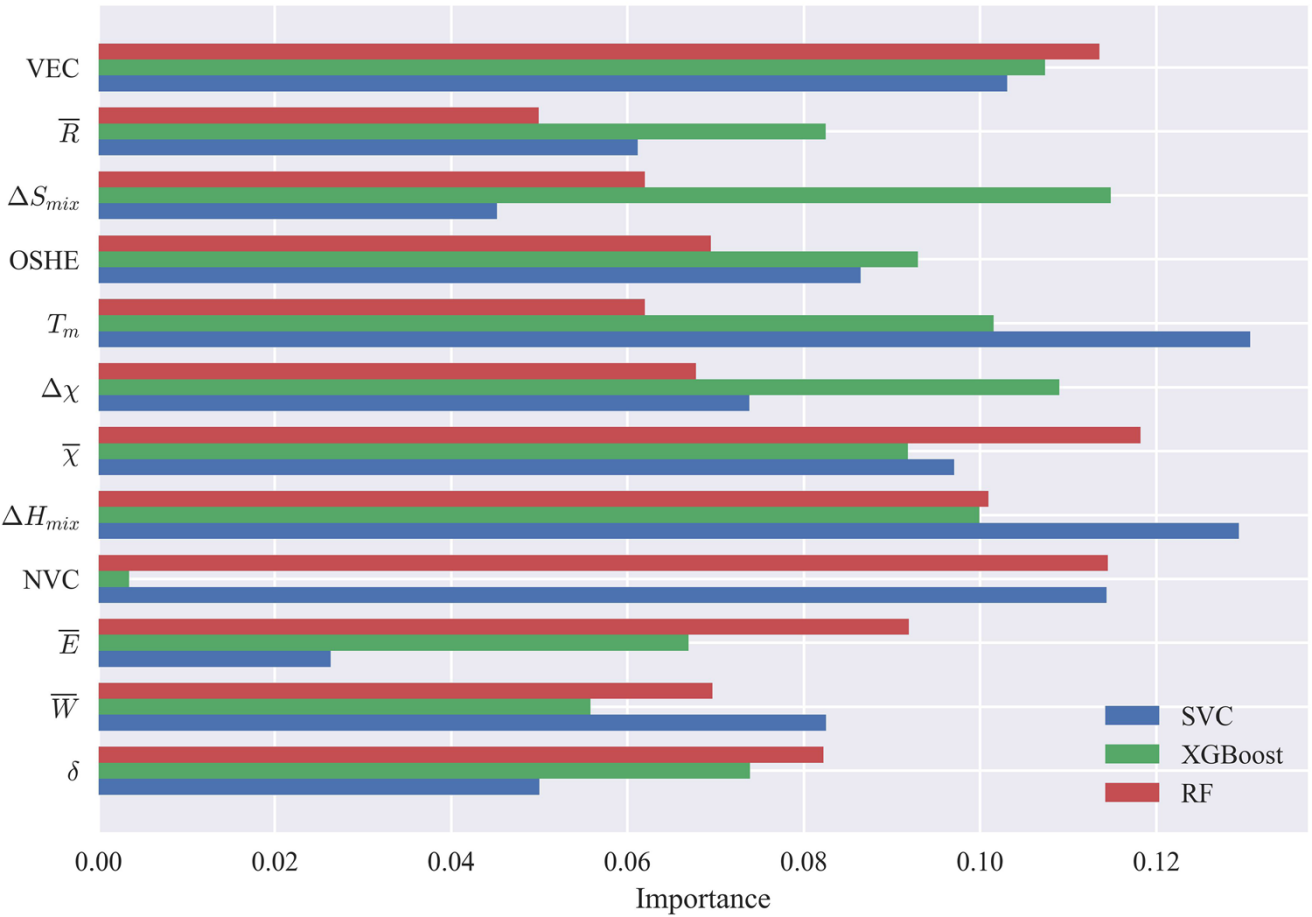
Feature importance analysis for predicting SS phases using RF, XGBoost, and SVC models. The figure illustrates the weight magnitudes of selected features in each model, highlighting the varying importance of features across different models. The results emphasize the model-specific nature of feature significance in high-entropy alloy phase prediction. XGBoost prioritizes mixing entropy and electronegativity deviation, RF considers average electronegativity, and SVC relies heavily on the melting point. Notably, the NVC and OSHE exhibit promising potential as predictive features for SS phases, providing new insights beyond the traditional focus on VEC and atomic size differences.

Fig. 4 provides a visual representation of how different selected features contribute to the accurate prediction of SS phases by the RF, XGBoost, and SVC models. Artificial Neural Networks (ANNs) are not included in this illustration because they are characterized by the combination of neurons with non-linear activations across multiple hidden layers, and are often considered complex and less interpretable and transparent compared to other models^75,76^. Essentially, the figure shows the weight magnitude of feature coefficients for each of these models. ML models learn a set of weights or coefficients associated with each feature, indicating the strength of the relationship between the feature and the correct prediction. By examining these weights, we can gain insights into the effects of each feature on the models under consideration. Features with higher weight magnitudes are deemed more important as they contribute significantly to the model’s predictions, while features with smaller weight magnitudes have a lesser impact. In the literature, various papers have highlighted different features as the most significant in predicting the phases of HEAs. For instance, refs^44,54,58,77^ identified VEC as the key feature, while refs^46,78^ emphasized the importance of atomic size difference, and refs^37,79^ focused on mixing enthalpy as the most important feature. However, upon analyzing the results, it becomes evident that the significance of these features in phase prediction varies considerably based on the specific ML model used and the types of phases present in the dataset. For instance, in the SVC model, the average melting point ($T_m$) emerges as the most crucial feature for achieving accurate predictions. However, in the case of RF, this feature does not hold the same level of importance. Conversely, the XGBoost model relies heavily on mixing entropy ($\Delta S_{mix}$) to accurately predict phases, yet this feature does not rank among the top five important features for the SVC model. Interestingly, the number of valence electrons (NVC) plays a significant role in the classification for both the RF and SVC models, yet its impact on classification by the XGBoost model is almost negligible. These disparities highlight the model-specific nature of feature importance in HEA phase prediction. These findings emphasize the need to carefully consider the choice of ML model when determining the importance of features in predicting HEA phases. Among all the models, the XGBoost demonstrated the highest accuracy, and its top features contributing to accurate predictions are the mixing entropy, electronegativity deviation ($\Delta \chi$), VEC, mixing enthalpy ($\Delta H_{mix}$), and $T_m$. Conversely, for the RF model, the most influential feature for accurate phase prediction is the average electronegativity(${\overline{\chi }}$), followed by the NVC, VEC, mixing enthalpy, and the total energy calculated by DFT (${\overline{E}}$). In the case of SVC, as mentioned earlier, $T_m$ emerges as the most important feature, followed by the mixing enthalpy and then the NVC. Additionally, VEC and outer shell electrons(OSHE) are also deemed important features of the SVC model. It is noteworthy that the NVC and OSHE show promising potential for predicting phases in solid solutions, despite receiving limited attention in the existing literature. While the focus in the literature has been predominantly on VEC and atomic size differences, the findings in this work demonstrate that different ML models value different features. This indicates that there might be additional features that have been overlooked in previous studies but could be extremely useful for accurate phase prediction in HEAs.

**Figure 5:**
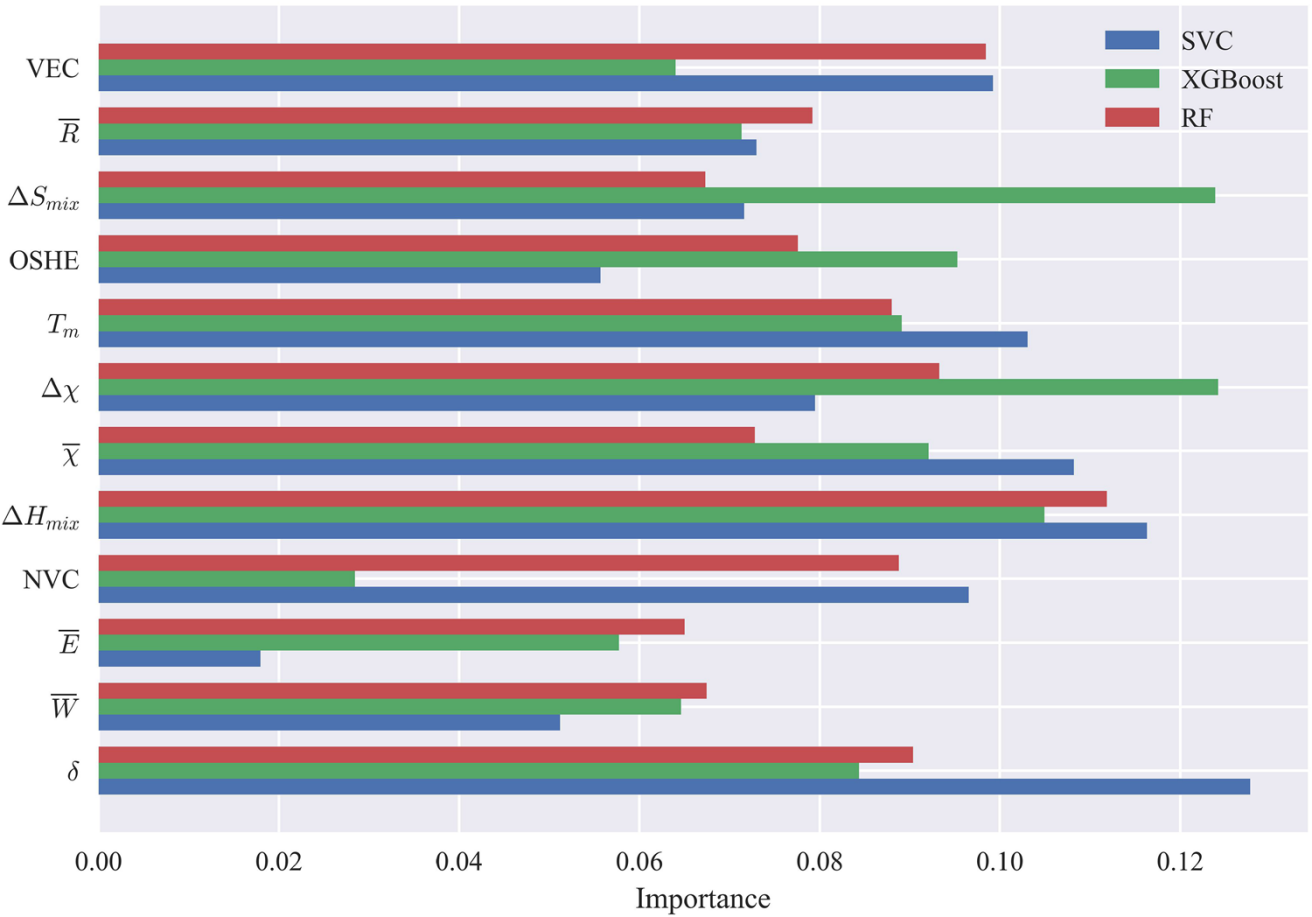
Feature importance analysis for predicting SS phases and their combinations with IM and AM phases using RF, XGBoost, and SVC models. The figure illustrates the changes in feature importance when considering the inclusion of IM and AM phases in the prediction analysis. Notably, electronegativity deviation gains importance in RF, indicating its relevance in accurately predicting the diverse range of phases. Atomic size difference experiences a notable shift in importance in SVC, emphasizing its critical role in classifying complex phase compositions. Mixing entropy, mixing enthalpy, and electronegativity deviation contribute significantly to accurate predictions by XGBoost, aligning with the importance of these features in predicting SS phases.

When considering the inclusion of IM and AM phases in the prediction analysis, there are notable changes in the importance of certain features. Fig. 5 provides an illustration of the feature importance for ML models when predicting all phases, including FCC, BCC, BCC+FCC, IM, BCC+IM, FCC+IM, BCC+IM, BCC+FCC+IM, AM, BCC+AM, FCC+AM, and BCC+FCC+AM phases. One significant change is observed in the importance of the (${\overline{\chi }}$) feature in the RF model, which was previously crucial for SS prediction. In the case of predicting all phases, the importance of ${\overline{\chi }}$ diminishes significantly, and instead, the importance of $\Delta \chi$ rises. This change indicates that electronegativity deviation becomes a more influential factor in accurately predicting the diverse range of HEA phases. Similarly, the atomic size difference feature ($\delta$) in the SVC model experiences a notable shift in importance when IM and AM categories are included. Its significance almost increases by a factor of 3, highlighting its importance in predicting all phases. This change suggests that atomic size difference plays a more critical role in accurately classifying the complex phase compositions encountered in HEAs when IM and AM phases are present. Overall, mixing entropy and mixing enthalpy, along with electronegativity deviation, contribute significantly to the accurate predictions made by the XGBoost model. This finding aligns with the importance of mixing entropy and enthalpy in predicting SS phases. Similarly, in the RF model, mixing entropy and enthalpy remain crucial, but the NVC becomes more important than electronegativity deviation. For the SVC model, atomic size difference accounts for approximately 13 percent of the prediction weight, surpassing other features. Mixing enthalpy and average electronegativity also retain their importance. It is worth noting that mixing enthalpy demonstrates a high degree of significance for all ML models and appears to be a critical factor for accurately predicting high-entropy alloy phases. Additionally, as previously mentioned, features such as NVC and OSHE can be utilized more frequently, as depicted in the figure, highlighting their importance in accurately predicting the diverse range of high-entropy alloy phases.

### Insight into phase formation by feature correlation

**Figure 6:**
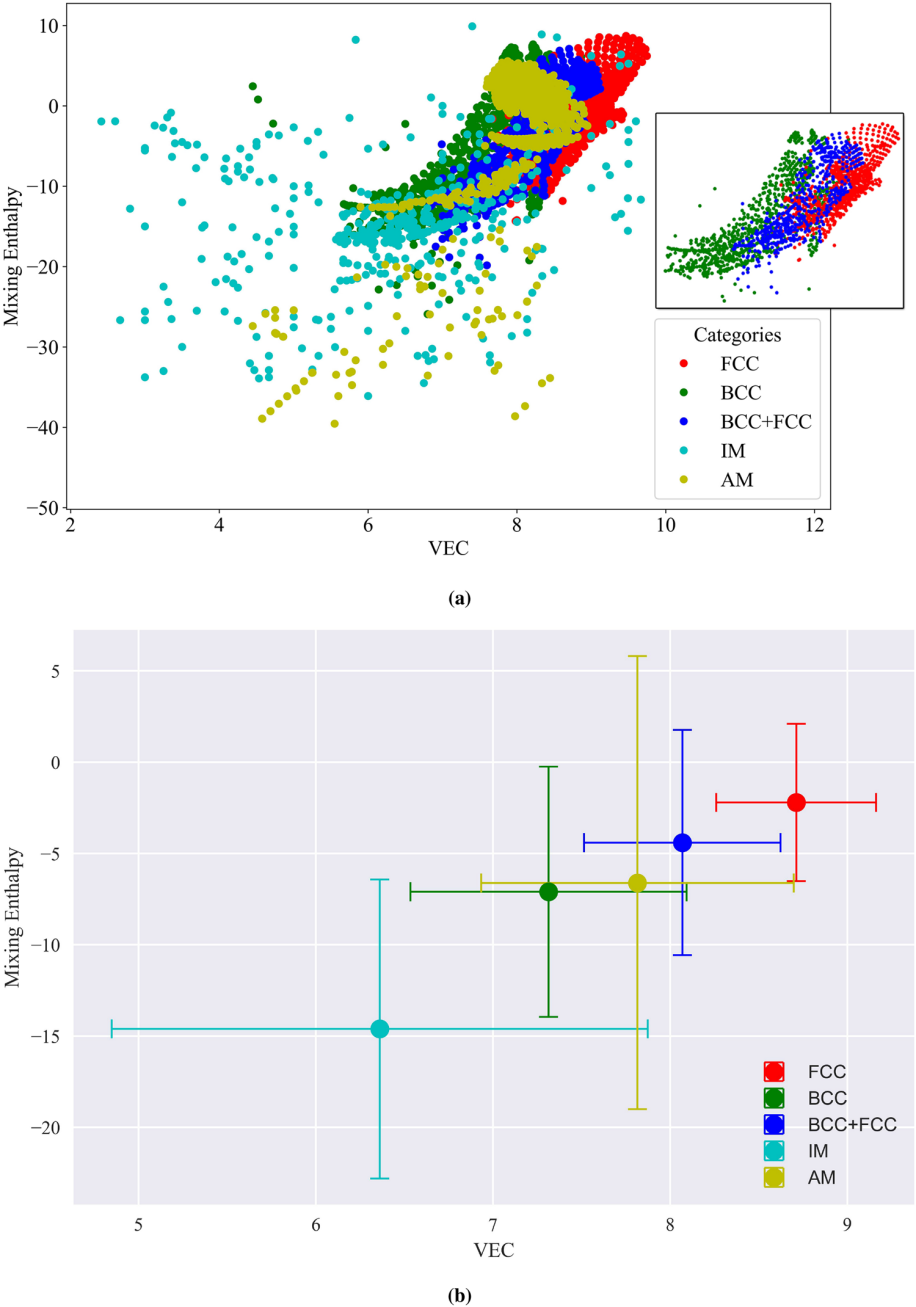
Variation of VEC and mixing enthalpy for different phases in high-entropy alloys. 6a depicts the relationship between VEC and mixing enthalpy for FCC, BCC, BCC+FCC, IM, and AM phases. IM and AM phases show less sensitivity to changes in VEC and mixing enthalpy compared to the distinct trend observed in the SS phases. 6b presents mean VEC and mixing enthalpy values, along with their standard deviations, for the mentioned phases.

Analyzing feature correlation is an important step in understanding the relationships between different features in a dataset. It allows us to explore how variables are related to each other and identify potential dependencies or patterns that can aid our understanding of the underlying physics. In the context of this study, exploring the correlation between VEC and mixing enthalpy is of particular interest. Understanding the correlation between VEC and mixing enthalpy is crucial for several reasons. Firstly, as discussed in the previous section, the current results point out the importance of VEC and mixing enthalpy in accurately predicting the phases of HEAs. Secondly, it helps in the assessment of the influence of electronic structure on the thermodynamic properties of HEAs. Fig. 6a shows the variation of VEC and mixing enthalpy for FCC, BCC, BCC+FC, IM, and AM phases. As observed in the figure, the IM and AM phases exhibit less sensitivity to changes in VEC and mixing enthalpy. On the other hand, the trend of the SS phases is clearly discernible. This distinct trend in the SS phases is likely the reason why the algorithms perform well in accurately categorizing them (BCC, FCC, BCC+FCC). Fig. 6b presents the mean values of VEC and mixing enthalpy, along with their corresponding standard deviations, for the phases under consideration. Fig. 6b reveals interesting insights regarding the VEC and mixing enthalpy values for different phases. The FCC phase exhibits the highest VEC, with a mean value of 8.71, indicating a larger number of VEC. On the other hand, the IM phase has the lowest VEC, with a mean value of 6.36, suggesting lower concentrations of the electrons in the valence band. For the BCC phase, the mean VEC value is around the middle range between FCC and IM, specifically 7.31, reflecting an intermediate VEC value. Similarly, the dual phase, consisting of both BCC and FCC structures, exhibits a mean VEC value of 8.06, which falls between the mean values of the individual BCC and FCC phases. The AM phase demonstrates a mean VEC value close to that of BCC, but slightly higher, measuring 7.81. This indicates a comparable number of valence electrons concentration to the BCC phase. Furthermore, the standard deviation of VEC values provides insights into the sensitivity of each phase to this feature. The FCC phase has the lowest standard deviation of 0.45, indicating that the VEC values for this phase are more consistent and less spread out from the mean. Conversely, the IM phase exhibits the highest standard deviation of 1.51, suggesting more variability in the VEC values and lower sensitivity to this feature. Fig. 6b also provides insights into the mean values of mixing enthalpy for different phases. The FCC phase exhibits the highest mean mixing enthalpy value, measuring -2.2. On the other hand, the IM phase has the lowest mean mixing enthalpy value, with -14.6, indicating a significantly lower mixing enthalpy for this phase. For the BCC phase, the mean mixing enthalpy value is -7.1, while for the dual-phase of BCC and FCC, the mean mixing enthalpy value is -4.4. The AM phase falls in between these values, with a mean mixing enthalpy value of -6.6. The standard deviation of mixing enthalpy values is considerably large, measuring 12.4. This indicates a significant variation in mixing enthalpy values among the different phases. However, the FCC phase has the lowest standard deviation of 4.3, suggesting that the mixing enthalpy values for this phase are more consistent and less spread out from the mean.

**Figure 7:**
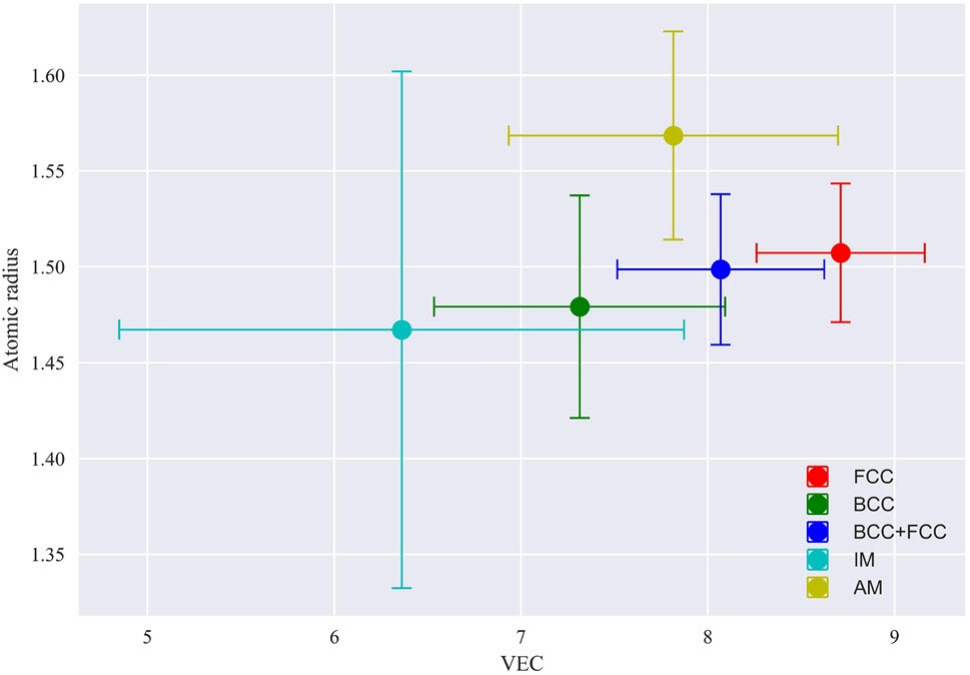
The correlation between VEC and atomic radius in HEAs. Notably, the atomic radius of AM HEAs stands out with its higher values, providing a key discriminative factor for distinguishing AM phases from solid solutions within the HEA spectrum.

As depicted in Figure 6b, it becomes evident that the mixing enthalpy values for AM and BCC categories are closely aligned. Consequently, using mixing enthalpy alone might not serve as an effective discriminator between the formation of BCC and AM phases within HEAs. In contrast, our findings indicate that, in this scenario, the atomic radius emerges as a valuable distinguishing factor as shown in Fig. 7. Noteably, When the atomic size exceeds a critical threshold of 1.54, the likelihood of AM phase formation significantly increases. Conversely, solid solutions tend to cluster around an average atomic radius of approximately 1.5, with a standard deviation of 0.03.

## Discussion

In the past decade, phase prediction in HEAs relied on extrapolation and computationally intensive first principle methods. Recently, data-driven approaches have gained popularity due to their excellent generalization capabilities. Generalization is generally more reliable than extrapolation^80^. However, the effectiveness of data-driven methods relies heavily on the size, quality, and diversity of the training data. Despite efforts to improve these aspects in the context of HEAs, creating highly appropriate data for ML prediction remains a challenge.

This paper introduces a data-driven method that leverages ML models to predict phase stability in HEAs. To ensure robustness, this study employs a vast dataset, carefully compiled from various references, consisting of 11,252 experimental observations. To ensure the accuracy and reliability of the analysis here, we implemented a rigorous data-cleaning process consisting of multiple steps, such as handling missing values, removing duplicates, standardizing formats, dealing with outliers, and resolving inconsistencies. Following meticulous data cleaning, we obtained a comprehensive dataset comprising 5,692 records with 50 different elements. The significance of single, dual, or triple-phase HEAs, particularly those with IM or AM phases, has garnered increasing attention in recent studies due to their specific applications^27,37,67,68,72,73^. Despite this significance, no ML model or dataset has adequately addressed the classification of them in the context of HEAs thus far. To address this limitation, a dataset encompassing all possible phases in HEAs, including SS, IM, AM, and their various combinations was created. By doing so, we aim to provide valuable insights into the prediction of these complex phases, offering a more complete and nuanced understanding of phase formation in HEAs. Nevertheless, an inherent challenge in the dataset lies in the unequal number of records across each phase category, posing potential biases for classification models. To mitigate this issue and ensure a balanced representation, we implement data augmentation techniques. This strategic approach results in a more equitable distribution, with each phase category containing approximately 1500 records of HEAs. By addressing this class imbalance, we enhance the reliability and robustness of the considered ML models, thus bolstering the accuracy and effectiveness of the phase predictions for diverse categories of HEAs.

At first, we explore the composition trends and prevalence of certain elements that promote the formation of BCC, FCC, IM, and AM phases. The analysis in this paper reveals the significant influence of Al on the formation of both BCC and IM phases in the alloy. While Al generally acts as a BCC stabilizer in HEAs, we observe that a higher concentration of Al specifically promotes the occurrence of the IM phase. This could be attributed to the fact that a higher amount of Al leads to an increase in the lattice constant and crystal system distortion^81^. Additionally, the diverse microstructure morphology, ranging from cotton-boll shape, cauliflower, spinodal, petal to fishbone shape, contributes to an increased driving force for ordering and the creation of IM phases^82^. Interestingly, we identify that Ni can be an IM stabilizer; however, its higher concentrations increase the likelihood of achieving an AM or FCC structure. Ni emerges as the most frequent element in the FCC category and the second most frequent in the AM category. Its relatively small atomic radius aids in reducing lattice strains within FCC systems^3^. However, this small radius may also contribute to a higher overall atomic size difference, thereby favoring the formation of AM phases. Notably, Mn exhibits a strong stabilizing effect on AM phases, attributed to its significant atomic size mismatch with typical HEA elements^83^. Moreover, the presence of multiple valence states for Mn (2+, 3+, 4+, etc.) results in fluctuating valence electron density, which disrupts ordering and crystallization processes^84^. Additionally, Fe exhibits remarkable versatility as an element, with no distinct preference for a specific phase, as it can contribute to the formation of any phase. This adaptability can be attributed to several factors, including its lattice adaptability^85^, size^3^, and variable valence^86^. Due to these unique characteristics, Fe plays a crucial role in influencing the phase formation and microstructure of HEAs, contributing to their diverse range of properties and potential applications. Among other important FCC stabilizers are Co and Cu, whereas Cr, Ti, Nb, and Zr act as BCC stabilizers.

In this field, most studies have commonly focused on using well-known features for ML training. However, we emphasize the significance of down-selecting effective features to enhance the approach deployed in this paper. Thus, we initiate a process by creating a diverse pool of features derived from chemical composition, the properties of the constituent elements, and the thermodynamics of HEAs. After constructing a diverse pool of features, we conducted a systematic analysis to extract the most significant ones for ML training. As a result, we identified 12 key features that serve as inputs for the training and evaluation of ML models suitable for classification. We also considered a diverse pool of ML models suitable for classification. Among these models, four consistently emerged as top performers in terms of accuracy. These include XGBoost, RF, ANNs, and SVC. These models were selected based on their ability to effectively capture the complex relationships within the data and provide accurate predictions across various phase categories in HEAs. This rigorous model selection process ensures that our analysis is underpinned by the most effective combination of features and algorithms. Upon comparison, the tuned RF and XGBoost models achieved an unprecedented accuracy of 97% in predicting phases in solid solution HEAs. However, when including AM phases and their combinations with SS, the tuned ANNs demonstrated better performance, achieving 95% accuracy. The inclusion of IM phases and their combinations further decreased the accuracy to 89%, indicating that IM-related phases are less sensitive to features than the AM category. Importantly, our comprehensive analysis covered all possible phases, encompassing 11 distinct categories involving SS, IM, AM, and their various combinations. Among all the ML models used, XGBoost stood out as the top performer, achieving an accuracy of 86%. The results illustrate the diverse performance of different ML models across different categories and how increasing the number of categories affects overall accuracy. XGBoost consistently exhibits robust performance, especially in predicting all phases, whereas SVC shows comparatively weaker performance in the expanded categories. These findings underscore the significance of choosing an ML model tailored to the specific application and desired category predictions for accurate high-entropy alloy phase prediction.

We compare the performance of these models across different phase categories to assess their accuracy in predicting phase stability. The results reveal that the inclusion of IM phases had a more pronounced impact on accuracy compared to the inclusion of AM phases. However, both types of phases influenced the performance of the models to some extent. Notably, the XGBoost and RF models consistently outperformed the other models in terms of accuracy. These models achieved 89% accuracy in predicting all 11 phase categories, surpassing the results reported in previous studies. This underscores the potential of ML techniques in the rational and timely design of alloys for specific applications. Furthermore, we investigate the impact of synthetic data on the accuracy of the models. Interestingly, we find that the inclusion of synthetic data has minimal effect on accuracy, indicating the robustness of the models. This suggests that the ML models can be generalized to new and unseen data, even when synthetic data is introduced.

In the literature, various papers have highlighted the importance of feature selection and they introduced different features as the most significant in predicting the phases of HEAs. For instance, refs^44,54,58,77^ identified VEC as the key feature, while refs^46,78^ emphasized the importance of atomic size difference, and^37,79^ focused on mixing enthalpy as the most important feature. However, upon analyzing the results, it becomes evident that the significance of these features in phase prediction varies considerably based on the specific ML model used and the types of phases present in the dataset. The results reveal the promising influence of NVC, OSHE, $\Delta \chi$, and total energy calculated from DFT in achieving more accurate predictions for various categories. These features demonstrate their potential for enhancing the precision of phase prediction across different scenarios. Moreover, through feature correlation analysis, we gain insights into the reasons behind the variability in algorithms’ performance in different categories. We observe the excellent performance of algorithms in categorizing SS HEAs, as well as the lower sensitivity of IM and AM phases to changes in important features. Notably, the FCC phase displays the lowest standard deviation in VEC and mixing enthalpy values, indicating higher consistency and a narrower spread from the mean, leading to improved phase categorization. Conversely, the IM phase exhibits the highest standard deviation of 1.51, suggesting greater variability in VEC values and reduced sensitivity to this feature. For AM, the mixing enthalpy displays higher standard variation than IM.

Additionally, we analyze the ranges of VEC and mixing enthalpy (the most effective and well-known features) in which certain phases might appear. Notably, the VEC and mixing enthalpy ranges for the BCC and AM phases are closely situated (Fig. 6a), suggesting similarities in electron configurations and thermodynamic characteristics between these two phases. This proximity underscores the need to consider additional features to effectively distinguish between them. Furthermore, the dual-phase combination of FCC and BCC exhibits VEC and mixing enthalpy values that represent an intermediate between the individual FCC and BCC phases. The presence of IM phases has a noticeable effect on lowering the VEC and mixing enthalpy values, setting it apart from other phases within the dataset.

Mixing enthalpy and VEC have garnered significant attention in the context of phase stability in HEAS. Previous research, exemplified by Zhang et al.^87^, suggests that mixing enthalpy values within the range of -15 to 5 often leads to the formation of solid solutions. Similarly, Guo et al.’s work^88^ indicates that the solid solution propensity extends from -5 to 5. However, our study delves deeper into this aspect. We’ve found that when mixing enthalpy exceeds -10, it notably promotes the formation of solid solutions or AM. Conversely, mixing enthalpy values lower than -10 are indicative of non-solid solution phases, particularly IM. For mixing enthalpy values between 5 and -10, the solid solution can manifest in various forms: single-phase or dual-phase, occasionally coexisting with AM. To distinguish between these manifestations, we turn to another influential factor, VEC. VEC’s impact on the stability of solid solution HEAs is well-documented, as shown in the work by Guo et al.^88^. Their experiments convincingly illustrate that VEC values exceeding 8 promote the formation of FCC phases, while values lower than 6.87 tend to favor BCC phases. These findings have been corroborated by various ML studies, including those conducted by Singh et al.^89^ and Ren et al.^59^. To further scrutinize VEC’s influence across the dataset, we calculated mean VEC values along with standard deviations (Fig. 6b). For BCC, the mean VEC is 7.33 with a standard deviation of 0.8, while for FCC, it’s 8.7 with a standard deviation of 0.45. These statistics emphasize the significance of specific VEC ranges in solid solution phase formation. For instance, single-phase FCC typically materializes when VEC approaches  8.2. Within the VEC range of 7 to 8.2, the possibility of various phases coming into existence becomes evident. As VEC decreases within this range, the likelihood of FCC formation diminishes while the possibility of AM and BCC formation increases. Once VEC drops below 7.5, the probability of discovering FCC becomes nearly negligible, and single-phase BCC dominates (Fig. 6b). However, distinguishing between BCC and AM solely based on mixing enthalpy and VEC within the VEC range of 7 to 8.2 can be challenging. Both categories exhibit similar average values for mixing enthalpy and relatively small differences in VEC. Our findings emphasize the significance of atomic radius (size) as a more effective descriptor for this purpose. In particular, an average atomic size radius exceeding 1.54 strongly indicates the presence of the AM phase, whereas values falling below this threshold favor BCC dominance (Fig. 7). Furthermore, higher VEC values coupled with elevated Mixing enthalpy are indicative of FCC presence, whereas VEC values lower than 6.7 combined with mixing enthalpy below -10 provide strong evidence for the presence of IM phases. These insights collectively contribute to a comprehensive understanding of phase stability in HEAs.

## Methods

### Data processing

**Figure 8:**
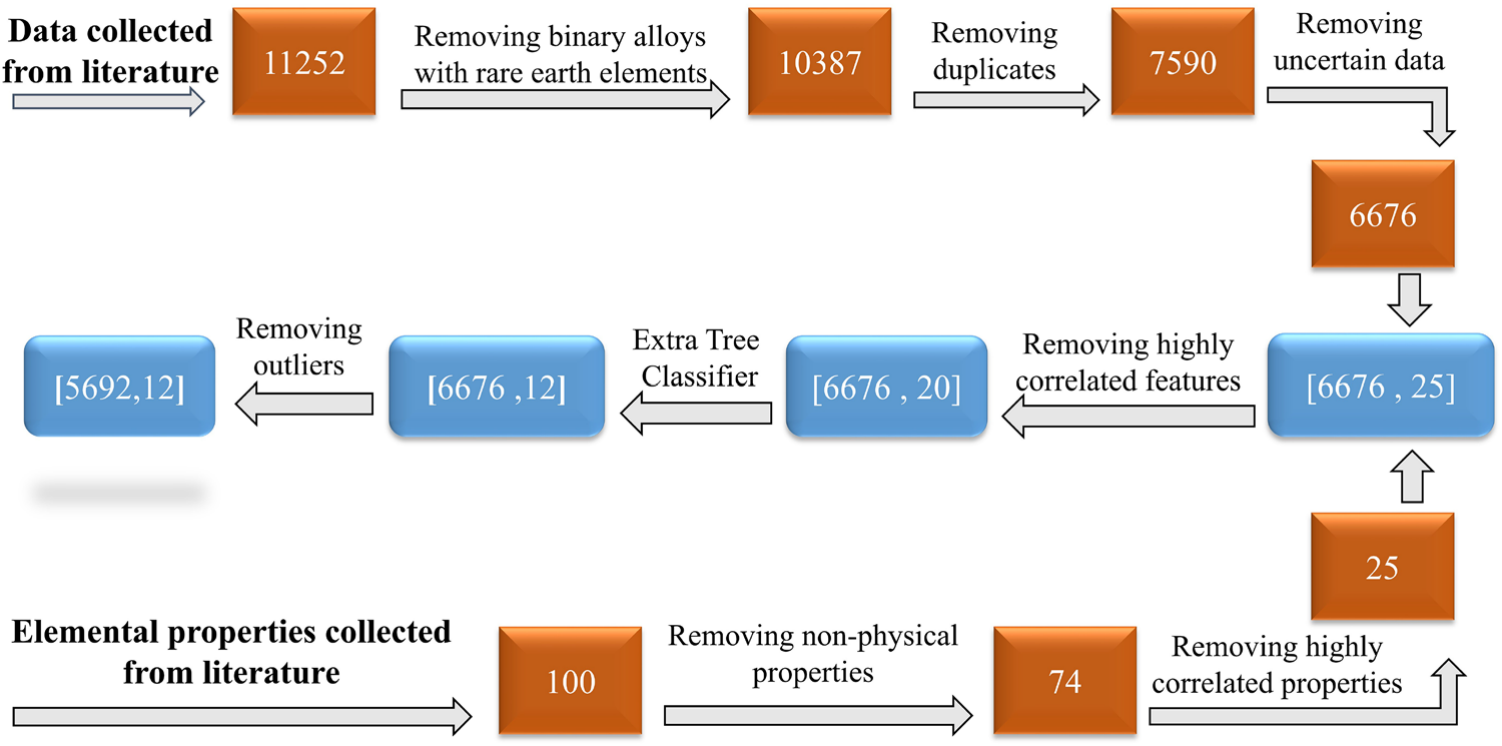
The process of data cleaning and generating features from literature.

The dataset utilized in this paper was sourced from various references^2,53–55,60,70,89–103^ encompassing 11 different categories and comprising a total of 11,252 records of HEAs. These categories include FCC, BCC, BCC+FCC, IM , BCC+IM, FCC+IM, BCC+IM, BCC+FCC+IM, AM, BCC+AM, FCC+AM, and BCC+FCC+AM phases. It is important to note that in this analysis, we have considered the single-phase B2 structure as BCC for consistency and classification purposes. To ensure data accuracy and avoid redundancy, we employed several data-cleaning steps. Firstly, we removed binary alloys containing rare earth elements from the dataset, as they can introduce complexities and unique behaviors. Next, we eliminated duplicate data that appeared in multiple sources, ensuring that each data point is represented only once. Additionally, we encountered about 900 records with the same composition but different reported phases. These variations can be attributed to differences in the fabrication process. To maintain a consistent dataset across all synthesis routes, we excluded these records as well. In the final step of data cleaning, we identified and eliminated data points with features that deviated significantly from the rest of the dataset, commonly known as outliers. By removing outliers, we aimed to focus the AI model’s learning on the majority of the data, which represents the underlying patterns and relationships. This process enhances the model’s accuracy and robustness in making predictions. Fig. 8 illustrates the data cleaning process we employed, illustrating the flow from data collection to outlier removal. The process of generating features will be explained in the next section. Also, Table 1 provides an overview of the cleaned data for each category, indicating the frequency of occurrence for each phase. The cleaned dataset, as summarized in Table 1, provides valuable insights into the frequency of occurrence for each phase category. It is noteworthy that the BCC structure is the most frequently mentioned in the literature, closely followed by the FCC structure. Additionally, the dual-phase structure combining both FCC and BCC is also commonly discussed^55,57,89,95^. Although the dataset includes alloys with varying numbers of components, ranging from three to ten, the majority (93%) of the data falls within the range of four to six components. Moreover, the alloys in the dataset consist of 50 different elements. However, certain elements, including Fe, Ni, Cr, Co, Al, Cu, Ti, Mn, V, Zr, Mo, Nb, Ta, and Zn, are more commonly used in the fabrication of HEAs.

**Table 1:** An overview of data organized according to their phase category and frequency of occurrence.

Phase category	Number of occurrence
BCC	1915
FCC	1500
BCC+FCC	1193
AM (AM)	951
IM (IM)	542
FCC+IM	164
BCC+IM	145
FCC+AM	116
BCC+AM	72
BCC+FCC+IM	44
BCC+FCC+AM	34

### Feature generation and selection

While the composition of HEAs provides valuable insights, it is not sufficient on its own to develop reliable and accurate ML models. To build robust models, additional features or descriptors are necessary to supplement the composition of data. Therefore, in this study, we recognize the importance of creating effective features and begin by constructing a diverse pool of features derived from chemical composition, a set of principles and formulas, and the properties of the constituent elements.

Mathematical equations play a crucial role in generating these features. We employ the rule of mixture, represented by the equation $X = \sum _{i=1}^{n} c_i(X_i)$, where $X_i$ denotes the elemental properties of each component, $c_i$ represents the composition of each component, and *n* corresponds to the number of components in the alloy. Another equation used is the deviation from the mean value, expressed as $\Delta X= \sqrt{\sum _{i=1}^{n} c_i(X_i-X_{\text {mean}})}$, where $X_{\text {mean}}$ denotes the mean value properties of all constituent elements. Additionally, we consider the percentage difference in elemental properties using the equation $\delta X= 100 \times \sqrt{\sum _{i=1}^{n} c_i\left( 1-\frac{X_i}{X_{\text {mean}}}\right) }$. Data on the available properties of each element or component, amounting to 100 properties, were gathered from^104^. Furthermore, we take into account other important features, such as the enthalpy and entropy of mixing, which can be estimated using equations 1 and 2, respectively.

1 $$\begin{aligned} \Delta H_{mix}= & {} 4\sum _{i=1, i\ne j}^{n} c_i c_j H_{ij} \end{aligned}$$

2 $$\begin{aligned} \Delta S_{mix}= & {} -R\sum _{i=1, i\ne j}^{n} c_i Ln c_i \end{aligned}$$

Equation 1 uses the mixing enthalpy $H_{ij}$ obtained from^105^ where $i_{th}$ and $j_{th}$ represent the elements, while constant *R* denotes the gas constant Additionally, new features can be calculated based on existing ones. For example, $\Omega$ introduced by^103^ is calculated as $\frac{T_m \Delta S_{\text {mix}}}{\left| \Delta H_{\text {mix}}\right| }$ and has shown potential for predicting solid solution formation. Another example is the geometrical parameter $\Lambda$, calculated as $\frac{\Delta S_{\text {mix}}}{(\delta R)^2}$^106^. The objective is to incorporate a comprehensive set of features that go beyond the composition of the data, enabling more accurate and robust predictions. However, using a large number of features in ML training can be computationally expensive and has the potential to limit the physical insights that can be gleaned from the data^107^. Therefore, feature selection is necessary to reduce model complexity and prevent overfitting, where the model performs poorly on new, unseen data. To perform feature selection, a three-step process was followed, as illustrated in Fig. 8. In the first step, non-physical elemental properties were eliminated from consideration. These properties were deemed irrelevant to the problem at hand and were thus removed from the feature set. Secondly, we identified highly correlated properties and features and removed them as well. However, Basic correlation analysis is insufficient for capturing the relationship between elemental properties and alloy phases, so a more sophisticated nonlinear approach is required to accurately model this relationship. In the third step, we employed the ExtraTreesClassifier algorithm from the scikit-learn library^108^. This algorithm fits multiple randomized decision trees on different subsets of the dataset and selects the most effective features for the final model.

After applying the feature selection process, we systematically selected 12 features from the pool of options suitable for the HEAs phase classification including VEC, OSHE, NVE, ${\overline{R}}$, $\delta$, ${\overline{W}}$, $\Delta S_{mix}$, $T_m$, ${\overline{\chi }}$, $\Delta \chi$, $\Delta H_{mix}$, ${\overline{E}}$. These features capture various aspects of the composition, elemental, thermodynamics, and physical characteristics of HEAs, providing a comprehensive set of descriptors to train the ML model effectively.

Anomaly detection is a crucial step in data analysis, as certain data points may deviate from the normal trend in a dataset, known as outliers or anomalies. These outliers have the potential to negatively impact the accuracy of predictive models^109^. In the context of high-entropy alloys, anomalies can arise due to measurement errors, human errors, mistakes in data collection, or rare but valid observations. To identify and isolate outliers in the dataset, we employed an unsupervised learning algorithm called Isolation Forest^110^. This algorithm is specifically designed for anomaly detection.

**Figure 9:**
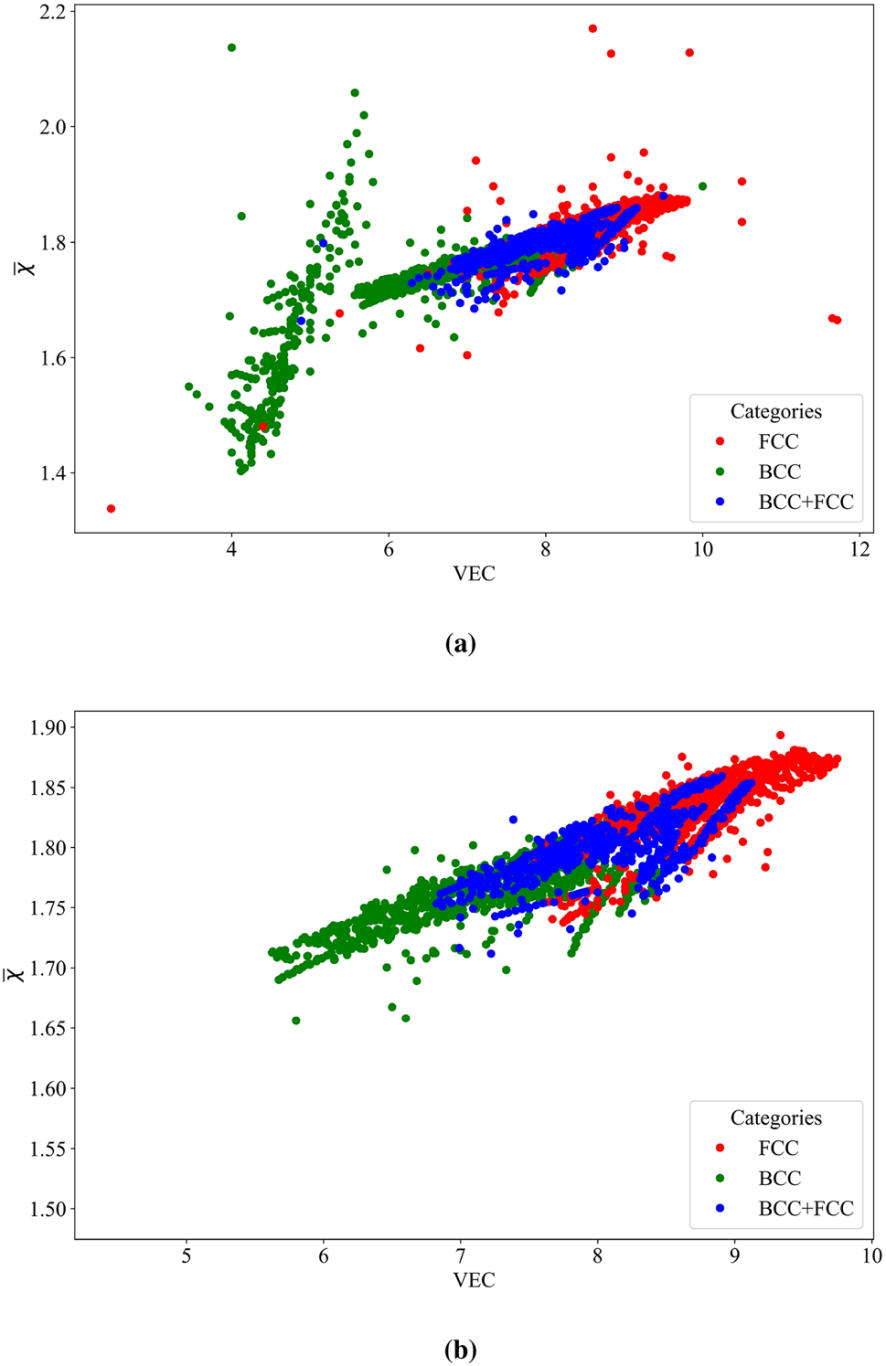
Impact of outlier detection and removal on solid solution classification. (**a**, **b**) point out the influence of detecting and eliminating outliers on the relationship between VEC and ${\overline{\chi }}$. In 9a all data points are included, displaying a less clear trend. However,9b exhibits the improved pattern after outlier identification and removal, resulting in a more distinct and identifiable relationship.

Fig. 9a and 9b illustrate the impact of detecting and removing outliers on the classification of solid solutions. The analysis focuses on the two most significant features for classification, average electronegativity (${\overline{\chi }}$) and valence electron concentration (VEC). In Fig. 9a, the relationship between VEC and average electronegativity is displayed for all data points of FCC, BCC, and dual-phase BCC+FCC HEAs. However, Fig. 9b demonstrates the improved trend after identifying and removing outliers. The updated pattern becomes more distinct and identifiable, leading to better performance of the ML model. This comparison highlights the importance of outlier detection and removal in enhancing the accuracy of the classification process for HEAs. By eliminating outliers, the underlying patterns and relationships in the data become more apparent, enabling more reliable predictions and insights.

Data preparation is an essential step before training an ML model. During this process, feature values are prepared, and one common technique is normalization. The normalization of feature values helps to scale the data by utilizing the mean value and standard deviation, as shown in Equation 3:

3 $$\begin{aligned} X = \frac{X_i - X_{\text {mean}}}{X_{\text {std}}} \end{aligned}$$

**Figure 10:**
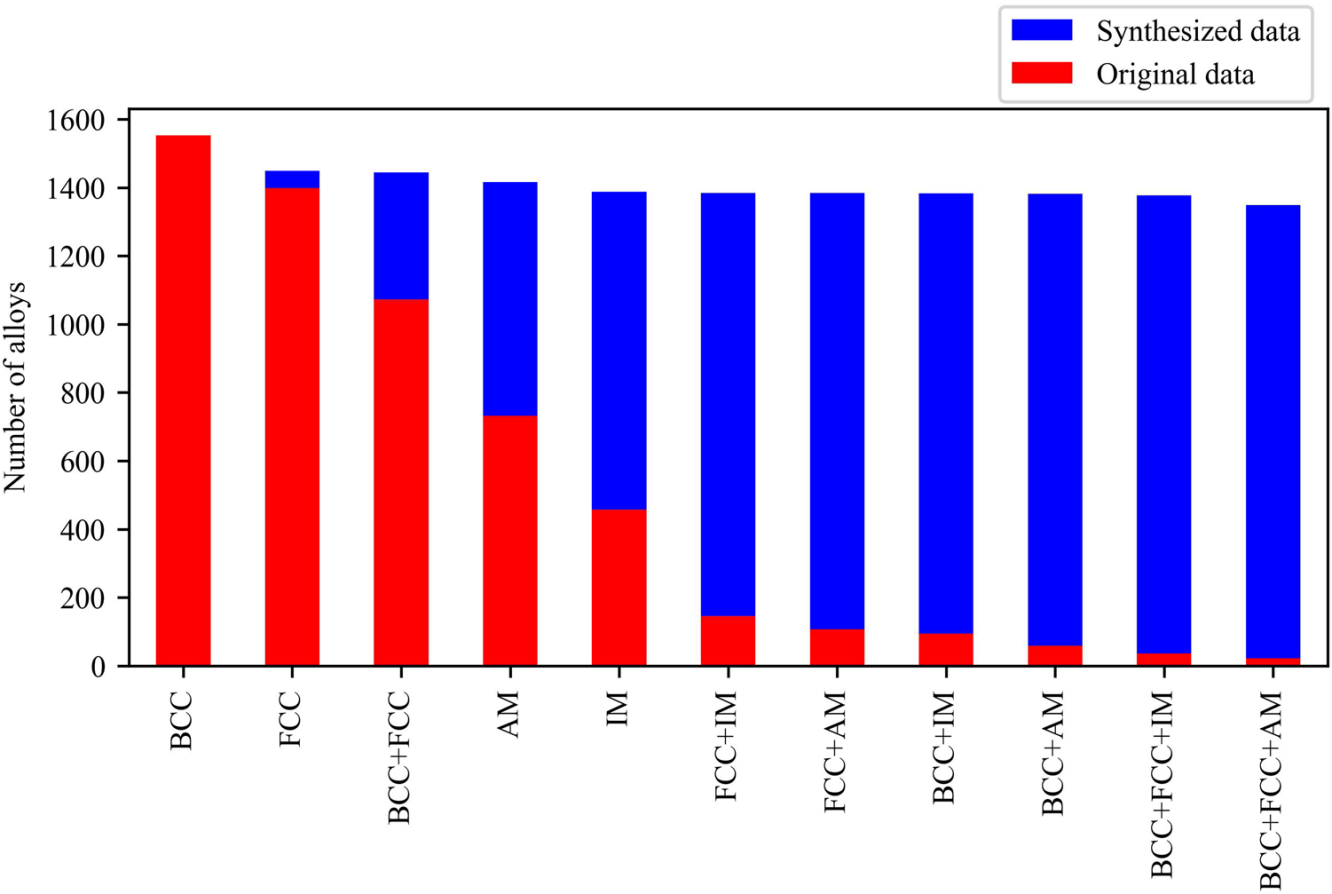
Impact of data generation on class balancing. presenting the distribution of alloys across different categories before and after the data generation process. Following the application of data generation techniques, it is evident that the number of records in each category has been equalized, effectively addressing the class imbalance concern.

In Equation 3, $X_i$ represents the feature value, $X_{\text {mean}}$ denotes the mean value of the data for that specific feature, and $X_{\text {std}}$ indicates the standard deviation of the data for that feature. Normalization helps to bring the features to a similar scale, which can improve the performance and convergence of ML models. ML models are typically trained and tested using three subsets of the dataset: the training, the validation, and the test dataset respectively. The training dataset is used to optimize the model’s trainable parameters. The performance of the model is monitored using the validation dataset to avoid overfitting or underfitting. Finally, the test dataset is used to evaluate the accuracy of the trained model^111^. In this particular work, due to the limited availability of sufficient data, the entire dataset was randomly divided into two subsets: the training dataset and the test dataset. The training dataset contains 90% of the total data, while the remaining 10% was allocated to both the test and validation datasets. At this point, the training dataset is highly imbalanced. ML models trained on imbalanced data tend to exhibit a bias toward predicting the larger class or classes. This often results in the smaller classes being disregarded entirely. Essentially, when class imbalance is present within the training data, ML models tend to over-classify the larger classes due to their high prior probability. This phenomenon of over-classification of larger classes is in direct opposition to what we aim to achieve, particularly since it’s frequently the case that predicting the rare phases, such as FCC+IM, accurately is of utmost importance. Therefore, it becomes crucial to address class imbalance during model training. In order To tackle this problem, the Adaptive Synthetic Sampling approach^112^ was used to generate synthetic data. Fig. 10 displays the number of alloys in each category before and after the data generation process. After applying the data generation technique, it can be observed that the number of records for each category is equal, thereby mitigating the class imbalance issue. This balanced dataset can now be used to train ML models more effectively, ensuring that all classes receive sufficient attention and avoiding the dominance of larger classes during training.

### Machine learning models

We explored several ML models for the classification of HEAS. The focus here has been on utilizing generated features to achieve accurate classification. Each of these ML models has its strengths and weaknesses, and their performance may vary depending on the specific dataset and problem at hand. By exploring and comparing these models, we aim to identify the most suitable one for accurately classifying HEAs based on the generated features. Accuracy is a widely recognized metric in ML for classification tasks. It quantifies the model’s ability to correctly predict the class labels (which are the phases in this study) of the samples in the validation dataset. It is calculated using the following formula:

$$\begin{aligned} accuracy(y, {\bar{y}})=\frac{1}{n_{samples}} \sum _{i=0}^{n_{samples-1}} x \left\{ \begin{matrix} x=1 \; if \; {\bar{y}}_i=y_i\\ x=0 \; if \; {\bar{y}}_i \ne y_i \end{matrix}\right. \times 100 \end{aligned}$$

where ${\bar{y}}_i$ represents the predicted value for the $i_{\text {th}}$ sample. $y_i$ represents the true (actual) value for the $i_{\text {th}}$ sample. $n_{\text {samples}}$ is the total number of samples in the validation dataset. In this formula, accuracy is calculated as the ratio of correctly predicted samples to the total number of samples in the validation dataset, expressed as a percentage. Essentially, it provides an estimate of how well the model is performing in classifying the samples into their respective phases. We used accuracy as a metric to evaluate and compare the ML models in this study. The following is a brief description of each ML model used.

#### SVC

SVC tries to find an optimal hyperplane in a high-dimensional space that can separate different classes of data. The hyperplane is selected in such a way that it maximizes the margin between the closest points from each class^113^. To achieve this, SVC solves a quadratic optimization problem by determining the Lagrange multipliers that define the hyperplane^114^. The data points that are closest to the hyperplane are referred to as support vectors^115^. It’s important to note that in the optimization problem, the data only appear in the form of inner products. To potentially enhance the representation of the data, we can map the data points to a higher-dimensional space called the feature space. This mapping is achieved through a transformation defined as follows:

4 $$\begin{aligned} x = (x_1, x_2,..., x_n) \rightarrow (\phi (x),..., \phi _N (x)) \end{aligned}$$

Here, the functional form of the mapping, denoted as $\phi$ is determined implicitly by selecting a suitable kernel function. By substituting the inner product with an appropriate kernel function ($K(x_i, y_j) = < \phi (x_i), \phi (y_j)>)$, the data can be effectively transformed into the feature space. This transformation enables even non-separable data points in the original input space to become linearly separable. Here $x_i$ and $x_j$ are the features of $i_{th}$ and $j_{th}$ samples. Various kernel functions are available for SVC, including linear, polynomial, radial basis function, and sigmoid^116^. In this study, we utilized the Support Vector Classifier (SVC) algorithm for classification, employing a 6-degree polynomial kernel ($K(x_i, y_j) = (\gamma (x_i^T y_j) + c)^6$). The parameter ’c’ in the kernel function serves as a free parameter that governs the influence of higher-order terms relative to lower-order terms within the polynomial function. By applying the polynomial kernel, the input data was transformed into a higher-dimensional feature space representation. This transformation involved raising the dot product of the input vectors to the power of 6 while incorporating the appropriate modifications from ’c’ and the parameter ’$\gamma$’. The resulting representation enabled the SVC model to capture complex relationships present in the data. In this study, values of ’c’ and ’$\gamma$ were carefully selected based on a systematic and automated method for hyperparameter optimization. The parameter ’c’ was set to 5.0, determining the balance between different polynomial terms within the kernel function. Similarly, ’$\gamma$’ was assigned a value of 1.0 to achieve optimal performance. It is important to note that SVC requires numerical output for training. As a result, label encoding was used to convert the categorical output into a numerical format. This transformation facilitated the utilization of SVC on the dataset, enabling us to effectively train (by train dataset)and evaluate the classifier (by test dataset). By leveraging the 6-degree polynomial kernel and employing label encoding to handle categorical output, this approach allowed us to explore intricate relationships within the data while ensuring compatibility with the SVC algorithm. The degree of the kernel, c, and $\gamma$ were optimized using grid search cross-validation.

#### Random forest (RF)

RF is an old ensemble learning algorithm used for supervised ML tasks, including classification. It is one of the most widely used ML algorithms. RF creates a large number of decision trees and combines their predictions to create a more accurate and robust model. Each decision tree is trained on a random subset of the training data and a random subset of the features, which helps to reduce overfitting and increase diversity. The final prediction is made by aggregating and averaging the predictions of all the decision trees^117^. The objective function of RF can be represented as $F(x) = \sum _{i=1}^{N} f_i(x)$ where *F*(*x*) represents the final prediction made by the RF model on input data point x. N is the number of decision trees in the ensemble. $f_i(x)$ represents the prediction made by the $i_{th}$ decision tree on input point to each tree. To train the RF model, the algorithm employs a technique called bootstrap aggregating or bagging. This involves creating multiple subsets of the original dataset through random sampling with replacement. Each subset, known as a bootstrap sample, is used to train an individual decision tree. For the specific case, 100 trees in the RF model were used to predict the phase. Each tree was trained independently on a bootstrap sample, with random feature selection performed at each node. The final prediction was obtained by aggregating the predictions of these 100 trees. This figure was obtained from grid search cross-validation. By employing an RF model with 100 trees, we leveraged the diversity and collective decision-making power of the ensemble, which often leads to improved accuracy and generalization performance. It is important to note that label encoding is still required when using the RF algorithm for this task. Label encoding converts categorical labels into numerical format, enabling compatibility with the RF algorithm for classification.

#### XGBoost

XGBoost^118^ is a powerful algorithm that leverages decision trees and gradient descent to optimize the objective function iteratively. By combining multiple weak learners (decision trees), XGBoost creates a strong learner that minimizes a loss function, such as binary cross-entropy for classification tasks. The algorithm also incorporates regularization techniques to prevent overfitting. Initially, XGBoost generates a model based on the training dataset. It then iteratively improves the model by placing greater emphasis on the training samples that were previously misclassified. This process continues until a consolidated model is achieved, where the weak models are combined using weighted majority voting^119^. To train the XGBoost algorithm, label-encoded training data is fed into the model. The input features are utilized to make predictions, resulting in the output $y_i$. As mentioned, gradient boosting is an iterative process that minimizes the objective function by sequentially adding weak learners to the ensemble. The objective function of XGBoost, as shown in Equation 5, consists of two components: the training loss function ($loss({\hat{y}}i, y_i)$) and the regularization term ($\sum {k}^{n} \omega (f_k)$).

5 $$\begin{aligned} F = \sum _{i}^{n} loss({\hat{y}}i,y_i)+ \sum {k}^{n} \omega (f_k) \end{aligned}$$

In Equation 5, *loss* represents the training loss function, which quantifies the discrepancy between the predicted output (${\hat{y}}_i$) and the actual output ($y_i$) for each data entry. The regularization term ($\omega$) helps prevent overfitting. $f_k$ denotes the output of each individual tree. During each iteration, XGBoost calculates gradients and Hessians for the loss function with respect to the predicted values. These gradients and Hessians provide information about the direction and magnitude of errors from the previous iteration, guiding subsequent updates to the model. XGBoost constructs decision trees in a level-wise manner, greedily determining the best splits for each tree node based on the gradients and Hessians. The learning rate is a crucial hyperparameter in XGBoost that controls the contribution of each weak learner to the overall ensemble. It governs the influence of each tree on the final prediction. In this study, this important hyperparameter was carefully tuned by grid search cross-validation for each training scenario to achieve optimal results.

#### ANNs

**Figure 11:**
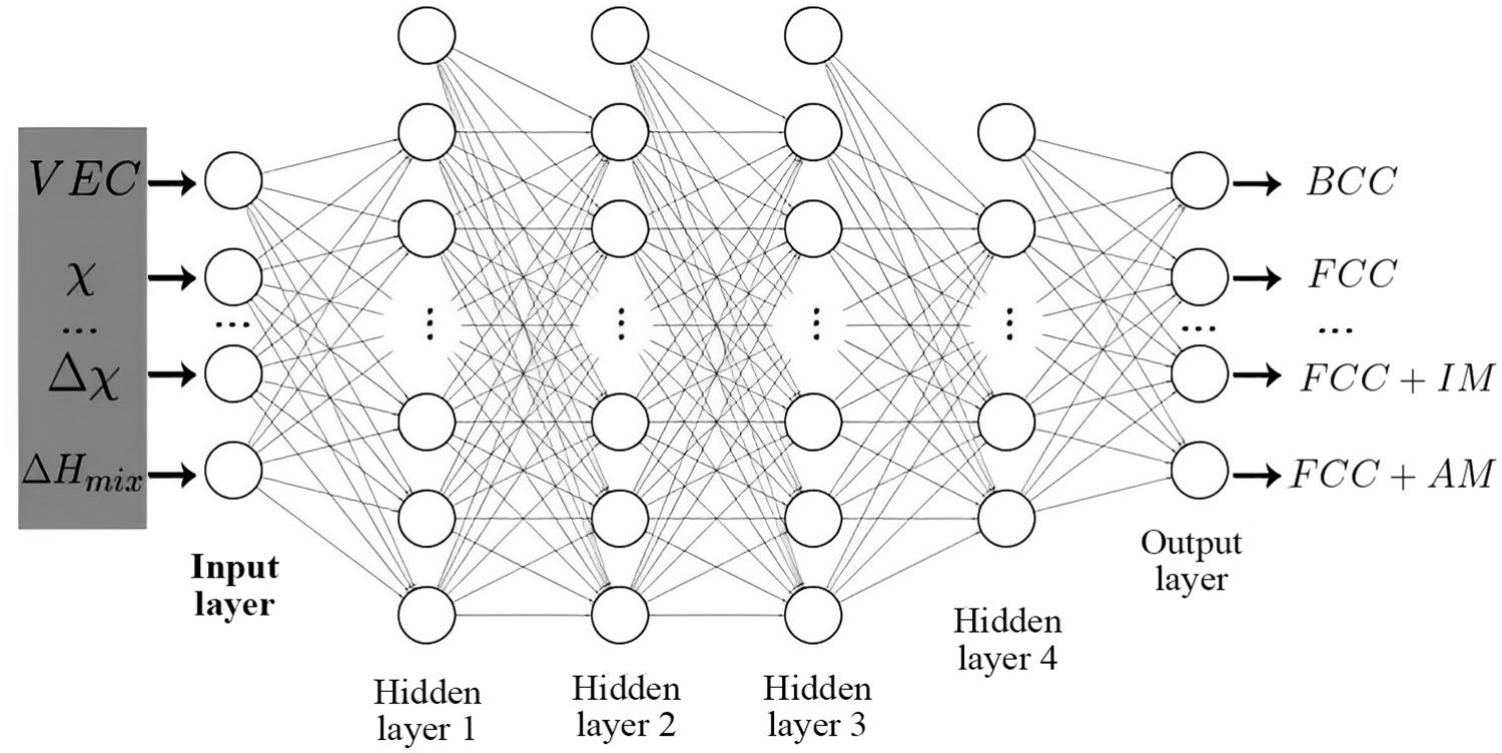
Architecture and training process of ANNs. It illustrates the structure of the neural networks employed in this study. ANNs consist of interconnected layers of processing nodes, known as neurons, which perform mathematical operations on input data to generate output values. 4 hidden layers with 160,161,80,16 neurons.

ANNs are composed of layers of interconnected processing nodes called neurons that perform mathematical operations on the input data to produce output values. Fig. 11 shows the architecture of NNs used in this study. The connections between the neurons have weights that are learned through a training process. The first layer consists of 12 neurons is equal to the number of input features as shown in Fig. 11. The last layer has neurons equal to the number of classes we want to classify. There are some hidden layers, whose number of neurons in these layers is influenced by the complexity of the data and the number of classes that need to be handled. The input features are passed forward to each neuron in the hidden layers, where they are multiplied by the respective fitted weights and transformed using an activation function. In this case, the Rectified Linear Unit activation function was used for hidden layers, and softmax was chosen for the final layer. At each hidden layer, a bias term is introduced and propagated along with the transformed values to the next layer. This process continues until it reaches the final output layer. In the final layer of the neural network, the output of the neural network is compared to the expected output using the loss function. For the classification task, the categorical cross-entropy loss function is used. Categorical cross-entropy is suitable when the true labels are one-hot encoded. Therefore, we transformed the categories into an array with integer encoding and then converted it into a one-hot encoded array. Equation 6 represents the softmax activation function, it applies the exponential to each input and then normalizes it between 0 and 1.

6 $$\begin{aligned} S(y_i) = \frac{e^{y_i}}{\sum e^{y_i}} \end{aligned}$$

Equation 7 presents the cross-entropy loss. where ${\widehat{y}}_i$ is the prediction by NNs and $y_i$ is the output. *N* is also the total number of data.

7 $$\begin{aligned} loss({\widehat{y}},y) = - \frac{1}{N} \sum y_i log({\widehat{y}}_i) \end{aligned}$$

For this study, the Artificial Neural Networks (ANNs) were built using TensorFlow^120^, in combination with the Keras^121^ library. The network is also tuned by ’RandomSearch’ tuner with KerasTuner^122^. We utilized the ADAM optimizer^123^ as the stochastic gradient descent algorithm with a specific learning rate of $5.0 \times 10 ^{-4}$. The ADAM optimizer had other parameters that were set to specific values, namely $\beta _1 = 0.9$, $\beta _2 = 0.9$, and $\epsilon = 10^{-7}$. During training, the neural network was trained for a total of 128 epochs, with a batch size of 32. These values were determined to be the optimal training parameters (hyperparameters) for this work.

## Data Availability

The data used for machine learning in this study is accessible in Github.com/Iman-Peivaste/ML_HEAs_Phase_Dataset.

## Code availability

The code used for machine learning in this study is open-source and accessible in Github.com/Iman-Peivaste/ML_Phase.
